# Cobertura e atraso vacinal nas coortes de nascidos em 2019 e 2020: inquérito domiciliar em Cubatão, Estado de São Paulo, Brasil

**DOI:** 10.1590/0102-311XPT089524

**Published:** 2025-05-19

**Authors:** Luciana Martins Rozman, Ana Marli Christovam Sartori, Daniela Banks, Rossana Veronica Mendoza Lopez, José Cássio de Moraes, Patrícia Coelho De Soárez

**Affiliations:** 1 Universidade São Judas - Campus Cubatão, Cubatão, Brasil.; 2 Hospital das Clínicas da Faculdade de Medicina da Universidade de São Paulo, São Paulo, Brasil.; 3 Secretaria de Saúde de Cubatão, Cubatão, Brasil.; 4 Instituto do Câncer do Estado de São Paulo, São Paulo, Brasil.; 5 Departamento de Medicina Social, Faculdade de Ciências Médicas da Santa Casa de São Paulo, São Paulo, Brasil.; 6 Departamento de Medicina Preventiva, Faculdade de Medicina da Universidade de São Paulo, São Paulo, Brasil.

**Keywords:** Cobertura Vacinal, Atraso Vacinal, Inquéritos Epidemiológicos, Vaccination Coverage, Vaccination Delay, Health Surveys, Cobertura de Vacunación, Retraso en la Vacunación, Encuestas Epidemiológicas

## Abstract

O Programa Nacional de Imunizações tem enfrentado recentemente a redução no alcance das metas preconizadas de cobertura vacinal para as vacinas constantes do calendário nacional infantil. Em Cubatão, município do Estado de São Paulo, os dados oficiais do Sistema de Informações do Programa Nacional de Imunizações apontam que a cobertura vacinal em crianças apresentou declínio acentuado. O objetivo deste estudo foi estimar a real cobertura vacinal em menores de 2 anos, avaliar se as doses estão sendo aplicadas em momento oportuno e identificar fatores associados ao atraso vacinal por meio de inquérito domiciliar em residentes de Cubatão nascidos em 2019 e 2020. O município foi dividido em cinco estratos socioeconômicos, e o tamanho da amostra foi de 450 crianças. A cobertura vacinal aos 12 meses foi de 86,9%; e aos 24 meses, de 82,6%. Para o esquema vacinal completo, apenas 25,7% das crianças foram vacinadas sem atraso. Entre os fatores associados ao atraso vacinal aos 24 meses estão escolaridade da mãe e receber benefício do Bolsa Família. Embora a cobertura vacinal seja maior do que a observada no inquérito nacional, o atraso para aplicação das doses é um problema de saúde pública em Cubatão, o que exige educação dos profissionais de saúde da ponta e da população sobre a importância da vacinação em tempo oportuno.

## Abstract (en)

The Brazilian Immunization Program has recently struggled to meet the recommended vaccination coverage rates for childhood vaccines included in the national schedule. In Cubatão, a municipality in the state of São Paulo, official data from the Information System of the Brazilian Immunization Program indicate a sharp decline in children’s vaccination coverage. The study aimed to estimate the actual vaccination coverage in children under 2 years old, assess whether doses are administered on time, and identify factors associated with vaccination delays using a household survey with Cubatão residents born in 2019 and 2020. The municipality was divided into five socioeconomic strata, and the sample included 450 children. Vaccination coverage at 12 months was 86.9%, and 82.6% at 24 months. Only 25.7% of children received the full vaccination schedule without delays. Factors associated with the delay in vaccination at 24 months included the mother’s educational level and receiving Bolsa Família benefits. Although vaccination coverage is higher than that in national surveys, delayed vaccination is a public health issue in Cubatão, requiring better education of healthcare professionals and the population on the importance of timely vaccination.

## Resumen

El Programa Nacional de Inmunización de Brasil ha enfrentado recientemente una reducción en el logro de los objetivos de cobertura de vacunación recomendados para las vacunas incluidas en el calendario infantil nacional. En Cubatão, un municipio del estado de São Paulo, los datos oficiales del Sistema de Información del Programa Nacional de Inmunización indican que la cobertura de vacunación en ese municipio en niños tuvo una gran disminución. El objetivo de este estudio fue estimar la cobertura real de vacunación en niños menores de 2 años de edad, evaluar si se están aplicando las dosis en un momento oportuno e identificar los factores asociados con el retraso de la vacuna mediante una encuesta de hogares con residentes de Cubatão para los nacidos en 2019 y 2020. El municipio se dividió en cinco estratos socioeconómicos; y la muestra contó con 450 niños. La cobertura de vacunación a los 12 meses fue del 86,9%; y a los 24 meses, del 82,6%. Para el calendario de vacunación completo, solamente el 25,7% de los niños estaban vacunados sin retraso. Entre los factores asociados con el retraso en la vacunación a los 24 meses destacan el nivel de estudios de la madre y el recibimiento del beneficio Bolsa Familia. Aunque la cobertura de vacunación es superior a la observada en la encuesta nacional, el retraso en la aplicación de las dosis es un problema de salud pública en Cubatão y requiere una capacitación a los profesionales de la salud y a la población sobre la importancia de la vacunación oportuna.

## Introdução

Desde a criação do Programa Nacional de Imunizações (PNI) em 1973, a imunização infantil é uma das políticas de saúde mais bem-sucedidas no Brasil, alcançando cobertura próxima da universalidade para crianças menores de 5 anos de idade. Devido ao seu caráter universal e gratuito e ao número e qualidade das vacinas oferecidas à população, o PNI é considerado um dos melhores programas de imunização do mundo ^1^ ^,^ ^2^. No entanto, observa-se que desde 2016 as coberturas vacinais têm diminuído ^3^ ^,^ ^4^ ^,^ ^5^, o que compromete a imunidade coletiva e aumenta o risco de ressurgimento de doenças até então controladas ou eliminadas ^6^ ^,^ ^7^.

Alguns fatores têm sido apontados para explicar essa redução, como a própria complexidade do PNI com desabastecimento parcial de alguns produtos; problemas operacionais para a execução adequada da vacinação; falta de capacitação dos profissionais de saúde; horário restrito das salas de vacina e subfinanciamento da atenção básica ^4^. Além disso, o desconhecimento da importância da vacinação, a hesitação em vacinar, as falsas notícias sobre vacinas também têm contribuído para esse cenário ^8^. Agravando esta situação, a pandemia de COVID-19 teve impacto negativo na cobertura, com redução de 18% para poliomielite e pentavalente e de 27% para tríplice viral ^9^.

Em Cubatão, município com 112.476 habitantes, localizado na Baixada Santista, Estado de São Paulo, a queda da cobertura foi acentuada entre 2016 e 2022. Exceto pela vacina BCG, nenhuma vacina para menores de um ano alcançou a meta de cobertura nesse período. A cobertura da vacina pentavalente, por exemplo, reduziu de 86,7% em 2016 para 48,3% em 2022.

Uma limitação do cálculo de cobertura vacinal baseada nos dados oficiais é considerar apenas as doses aplicadas e registradas em relação à população estimada ^10^. Tal fato impede a identificação de áreas ou grupos populacionais com situação vacinal inadequada, visto que essas diferenças não são reveladas pela média ^11^ ^,^ ^12^. Outra limitação é que, embora forneçam informação sobre o estado vacinal atualizado, os cálculos de cobertura vacinal não revelam em que momento a vacina foi administrada. Dessa maneira, algumas crianças podem ser consideradas em dia com o esquema vacinal, mesmo que alguma vacina tenha sido administrada com atraso em relação ao recomendado ^13^ ^,^ ^14^ ^.^

A realização de inquérito domiciliar de cobertura vacinal permite estimar a real proporção de crianças vacinadas, localizar áreas com diferentes situações, avaliar as desigualdades sociais associadas e investigar os fatores que influenciam a cobertura vacinal ^11^.

Embora existam no Brasil inquéritos vacinais que forneçam informações úteis sobre o estado de vacinação ^15^ ^,^ ^16^ ^,^ ^17^, raros são os estudos que avaliam os atrasos vacinais até que as crianças completem o esquema vacinal ^4^ ^,^ ^18^, além disso, não há do nosso conhecimento, inquérito vacinal realizado na Baixada Santista.

Cubatão conta com um importante polo industrial e está situado próximo ao Porto de Santos, maior da América Latina, o que resulta em intensa circulação de pessoas de diversas regiões. A redução da cobertura vacinal em um município com essas características justifica a realização de inquérito domiciliar.

O objetivo deste estudo é estimar por meio de um inquérito domiciliar a cobertura vacinal em menores de 2 anos de idade residentes em Cubatão, avaliar se as doses estão sendo aplicadas no momento oportuno sem atraso vacinal e identificar fatores associados ao atraso vacinal.

## Métodos

### Delineamento

Trata-se de um inquérito de base populacional, realizado em Cubatão, entre março e julho de 2023.

O município foi dividido em estratos socioeconômicos considerando-se as condições de vida da população. Para essa divisão foram consultados profissionais de saúde do município com experiência no território. O estrato 1 foi classificado como o melhor; e o estrato 5, o de pior condição socioeconômica.

Foi realizado o georreferenciamento do endereço das 2.950 crianças nascidas vivas em 2019 e 2020 que em março de 2023 teriam entre 39 e 50 meses de idade. Para obtenção desses dados foi utilizado o Sistema de Informação sobre Nascidos Vivos (SINASC). Em seguida, foram formados, em cada estrato, conglomerados de setores censitários com 28 ou mais nascidos vivos. Um conglomerado poderia ser formado por um ou mais setores censitários desde que contíguos geograficamente e homogêneos socioeconomicamente. Em cada um dos estratos foram sorteados, por meio de amostragem sistemática, os conglomerados a serem incluídos na amostra.

As entrevistas foram feitas por entrevistadores treinados e registradas em formulário eletrônico (Google Forms; https://docs.google.com/forms). Durante as entrevistas, as cadernetas de vacinação foram fotografadas, para que os dados de doses aplicadas fossem posteriormente lidos e digitados por profissionais de saúde com experiência na área. As crianças que não tinham caderneta de vacinação no momento da entrevista não foram incluídas no estudo.

### Tamanho do estudo

Os parâmetros utilizados para o cálculo do tamanho da amostra foram: proporção esperada de crianças vacinadas = 0,70; nível de significância de 0,05; efeito do desenho = 1,5. Com base nesses valores, estimou-se o tamanho da amostra em 450 crianças ^19^. O tamanho da amostra foi dividido garantindo-se o mesmo número de crianças em cada estrato socioeconômico. Desse modo, cada estrato foi composto por 90 nascidos vivos.

### Variáveis

A coleta de dados das entrevistas domiciliares foi realizada por meio de um questionário padronizado com informações sobre o entrevistado, características da mãe da criança (escolaridade, trabalho remunerado), sexo da criança e características da família (renda, número de pessoas na residência, bens de consumo).

### Definições

(a) Cobertura vacinal para cada uma das vacinas incluídas no calendário nacional de vacinação - foi analisada para as seguintes vacinas: BCG, hepatite B ao nascer, pentavalente, vacina inativada da poliomielite (VIP), pneumocócica, meningocócica C, rotavírus, febre amarela, sarampo, caxumba e rubéola (SCR), varicela, hepatite A, 1º reforço de poliomielite e 1º reforço de difteria, tétano e coqueluche (DTP).

O cálculo da cobertura foi: número de crianças que completaram o esquema vacinal preconizado de uma vacina dividido pelo número de crianças incluídas no estudo multiplicado por 100.

(b) Esquema vacinal completo aos 24 meses ou até a idade da criança no momento da entrevista - uma dose de BCG e hepatite B ao nascer; três doses de pentavalente e da VIP; duas doses das vacinas pneumocócica, meningocócica C, rotavírus e SCR; uma dose das vacinas de febre amarela, varicela, hepatite A; e 1º reforço de pneumocócica, meningocócica, DTP e poliomielite.

(c) Esquema vacinal básico completo aos 12 meses ou até a idade da criança no momento da entrevista - uma dose de BCG, hepatite B ao nascer; duas doses de pneumocócica, meningocócica e rotavírus; três doses de pentavalente e de VIP; e uma dose de febre amarela.

O cálculo para cobertura do esquema completo aos 12 e 24 meses foi: número de crianças com esquema vacinal completo foi dividido pelo número de crianças incluídas no estudo multiplicado por 100.

(d) Classificação das doses recebidas ^15^ ^,^ ^20^ - (i) doses aplicadas: doses de cada uma das vacinas registradas na caderneta de vacinação, sem considerar idade ou intervalo entre as doses; (ii) doses corretas: doses recebidas com data de início conforme recomendação do PNI e intervalo adequado entre as doses; e (iii) doses com atraso vacinal: doses recebidas após idade limite para ser considerada correta, ou seja, 30 dias após o preconizado.

### Métodos estatísticos

Estatísticas descritivas foram calculadas para apresentar as variáveis qualitativas e quantitativas. Associação bivariada entre variáveis qualitativas foi realizada mediante o teste qui-quadrado de Pearson ou teste exato de Fisher.

Para o cálculo dos intervalos de 95% de confiança (IC95%), foram considerados os valores correspondentes ao efeito do desenho, ou seja, o efeito decorrente da utilização de uma amostra por conglomerados em múltiplos estágios. O efeito do desenho é o valor obtido pelo cálculo da razão da medida da variabilidade entre os conglomerados e a variabilidade entre os indivíduos interna a cada conglomerado, refletindo assim o grau de homogeneidade/heterogeneidade existente no inquérito, para cada uma das informações obtidas. A estimativa considera, também, os pesos correspondentes aos diferentes tamanhos de população em cada estrato. O peso amostral foi calculado da seguinte maneira:

$$wi=Ni\times \sum i\;ni÷\left(\sum i\;Ni\times ni\right)$$

no qual *Ni* e *ni* são os tamanhos da população e a amostra no estrato *i*, respectivamente.

O atraso vacinal foi considerado como variável desfecho. Considerou-se esquema sem atraso receber qualquer uma das doses anteriormente listadas, na idade e no intervalo estabelecido pelo PNI. Entendeu-se dose atrasada como a vacina aplicada com 30 dias ou mais da idade recomendada.

Estatísticas descritivas e testes exatos de Fisher e qui-quadrado foram utilizados para avaliar a associação entre o atraso vacinal e diversos fatores (aglomeração intradomiciliar - mais de três pessoas por dormitório, nível de consumo, renda da família, bolsa família, escolaridade da mãe, raça/cor da pele, frequentar creche e número de filhos vivos) de forma independente. Nível de significância (valor de p < 0,05) foi considerado para rejeitar as hipóteses nulas.

Estimou-se a razão de chances (OR, do inglês *odds ratio*) e IC95% por meio de regressão logística binária utilizando método *stepwise* com eliminação retrógrada de variáveis. Permaneceram no modelo final as variáveis com valor de p < 0,050. Todos os processamentos e análises estatísticas forma realizados por meio do software estatístico SPSS, versão 22 (https://www.ibm.com/).

### Aspectos éticos

O projeto foi aprovado pelo Comitê de Ética em Pesquisa da Universidade São Judas Tadeu (parecer nº 5.799.774), e desenvolvido com a anuência da Secretaria de Saúde de Cubatão.

## Resultados

Foram entrevistadas 407 famílias, correspondendo a 90,4% da amostra prevista. As perdas decorreram de recusa (n = 18), impossibilidade de realização da entrevista após duas visitas em dias e horários diferentes (n = 3) e impossibilidade de encontrar o número esperado de crianças mesmo após percorrer todos os domicílios dos conglomerados sorteados (busca ativa). As recusas, sempre que possível, eram substituídas por outra criança do conglomerado. Os estratos 1 e 2, com melhor condição socioeconômica, foram os que apresentaram as maiores perdas, com 81% da amostra prevista entrevistada (Tabela 1).

**Tabela 1:** Número e porcentagem de entrevistas realizadas e perdas em relação ao esperado, por estrato socioeconômico. Inquérito de cobertura vacinal, Cubatão, São Paulo, Brasil, 2023 (n = 407).

Estrato	Entrevistas realizadas	Perdas		Total esperado
		Recusas	Outras perdas	
	n (%)	n (%)	n (%)	n
1	73 (81,0)	2 (2,2)	15 (16,6)	90
2	73 (81,0)	6 (6,6)	11 (12,2)	90
3	85 (94,4)	5 (5,5)	0 (0,0)	90
4	83 (92,2)	5 (5,5)	2 (2,2)	90
5	93 (102,0)	0 (0,0)	0 (0,0)	90
Total	407 (90,4)	18 (4,0)	28 (6,2)	450

Aglomeração intradomiciliar (mais de três pessoas por dormitório) foi maior no estrato 5 (18,5%). No momento da entrevista, 43,3% das famílias recebiam Bolsa Família, 67,4% no estrato 5. Cerca de 30% das famílias do estrato 5 recebiam até R$ 1.000,00 como renda mensal. O estrato socioeconômico 1 foi o que apresentou maior proporção de famílias no nível de consumo A e B (30,8%). Nos estratos 4 e 5, a totalidade das famílias eram dos níveis C e D. Aproximadamente 50% das mães apresentaram Ensino Médio completo ou Superior incompleto (Tabela 2).

**Tabela 2:** Características das famílias segundo estrato socioeconômico. Inquérito de cobertura vacinal, Cubatão, São Paulo, Brasil, 2023.

Características das famílias	Estrato					
	1	2	3	4	5	Total
	n (%)	n (%)	n (%)	n (%)	n (%)	n (%)
Aglomerado intradomiciliar						
Sim	3 (4,1)	4 (5,5)	5 (5,9)	13 (15,7)	17 (18,5)	42 (10,3)
Recebe Bolsa Família						
Sim	14 (19,2)	23 (31,9)	33 (38,8)	44 (53,7)	62 (67,4)	176 (43,3)
Renda familiar mensal (R$)						
Até 1.000,00	5 (6,8)	15 (20,5)	12 (14,1)	26 (31,3)	30 (32,6)	88 (21,7)
1.000,00-2.999,99	23 (31,5)	22 (30,1)	38 (44,7)	33 (39,8)	48 (52,2)	164 (40,4)
3.000,00-5.000,00	20 (24,7)	19 (26,0)	19 (22,4)	16 (19,3)	3 (3,3)	77 (19,0)
Mais de 5.000,00	22 (30,1)	7 (9,6)	5 (5,9)	2 (2,4)	0 (0,0)	36 (8,9)
Nenhuma	1 (1,4)	0 (0,0)	1 (1,2)	1 (1,2)	5 (5,4)	8 (2,0)
Ignorado	2 (2,7)	10 (13,7)	10 (11,8)	5 (6,0)	6 (6,5)	33 (8,1)
Nível de consumo das famílias *						
A e B	20 (30,8)	12 (19,4)	7 (10,4)	0 (0,0)	0 (0,0)	39 (10,8)
C e D	45 (69,2)	50 (80,6)	60 (89,6)	76 (100,0)	90 (100,0)	321 (89,2)
Escolaridade da mãe						
Analfabeto ou Ensino Fundamental I incompleto	0 (0,0)	0 (0,0)	0 (0,0)	2 (2,4)	1 (1,1)	3 (0,7)
Ensino Fundamental I completo ou Fundamental II incompleto	1 (1,4)	3 (4,1)	4 (4,7)	7(8,4)	17 (18,5)	32 (7,9)
Ensino Fundamental II completo ou ensino médio incompleto	4 (5,5)	8 (11,0)	15 (17,6)	18 (21,7)	31 (33,7)	76 (18,7)
Ensino Médio completo ou Superior incompleto	32 (43,8)	42 (57,5)	33 (38,8)	52 (62,7)	40 (43,5)	199 (49,0)
Ensino Superior completo	32 (43,8)	14 (19,2)	8 (9,4)	3 (3,6)	2 (2,2)	59 (14,5)
Ignorado	4 (5,5)	6 (8,2)	25 (29,4)	1 (1,2)	1 (1,1)	37 (9,1)
Mãe com trabalho remunerado						
Sim	28 (38,3)	29 (39,7)	32 (37,6)	32 (38,5)	30 (32,6)	151 (37,2)
Raça/Cor da pele da mãe						
Branca	38 (52,0)	24 (32,9)	20 (23,5)	30 (36,1)	21 (22,8)	133 (32,7)
Parda	26 (35,6)	38 (52,1)	54 (63,5)	40 (48,2)	53 (57,6)	211 (52,0)
Preta	8 (11,0)	9 (12,3)	8 (9,4)	11 (13,3)	18 (19,6)	54(13,3)
Amarela	1 (1,37)	2 (2,7)	1 (1,2)	2 (2,4)	0 (0,0 )	6 (1,5)
Não declarado	0 (0,0)	0 (0,0)	2 (2,4)	0 (0,0)	0 (0,0)	2 (0,5)
Número de filhos vivos [média (mínimo e máximo)]	2 (1-4)	2 (1-7)	2 (1-6)	2 (1-5)	3 (1-9)	2,2 (1-9)
Sexo da criança						
Masculino	44 (60,3)	39 (53,4)	51 (60,0)	40 (48,2)	49 (52,7)	223 (54,8)
Frequenta creche						
Sim	61 (83,6)	61 (83,6)	63 (74,1)	61 (73,5)	67 (72,8)	313 (77,1)

* A classificação do nível de consumo das famílias se baseou nos critérios da Associação Brasileira de Empresas de Pesquisa ^41^.

Entre as 23 doses de vacinas previstas no calendário nacional de imunização, as metas de cobertura de doses aplicadas (90% para BCG e rotavírus e 95% para as demais) foram atingidas para todas as vacinas. No entanto, ao avaliar o momento da aplicação e a cobertura de doses sem atraso, apenas as vacinas BCG e contra a hepatite B alcançaram a meta. A cobertura de dose sem atraso diminui conforme a idade recomendada para aplicação da vacina aumenta. A proporção de crianças que tomou a dose de vacina com atraso variou de 3,2% para BCG a 44% para o primeiro reforço da vacina contra poliomielite (Tabela 3).

**Tabela 3:** Proporção de cobertura vacinal segundo vacina aplicada, avaliação de atraso na administração das doses (número de crianças com atraso e tempo médio de atraso) e cobertura vacinal sem atraso para cada imunobiológico do calendário nacional de vacinação para crianças nascidas em 2019-2020. Inquérito de cobertura vacinal, Cubatão, São Paulo, Brasil, 2023.

Imunobiológico	Cobertura vacinal	Crianças com atraso vacinal *	Cobertura vacinal, doses sem atraso	Tempo médio de atraso (dias)	DP do tempo (dias)
	% (IC95%)	n (%)	% (IC95%)		
BCG	99,3 (98,5-100,0)	13 (3,2)	96,1 (94,2-98,0)	77,7	117,3
Hepatite B	99,4 (98,6-100,0)	10 (2,5)	97,1 (95,4-98,7)	113,6	135,1
VIP (1ª dose)	99,6 (99,0-100,0)	39 (9,6)	89,3 (86,3-92,3)	83,3	122,6
VIP (2ª dose)	99,6 (99,0-100,0)	84 (20,6)	78,5 (74,5-82,5)	112,7	226,6
VIP (3ª dose)	98,7 (97,6-99,8)	123 (30,2)	69,4 (64,9-73,9)	117,1	203,5
Pentavalente (1ª dose)	99,6 (99,0-100,0)	41 (10,1)	89,5 (86,5-92,5)	81,5	124,3
Pentavalente (2ª dose)	99,6 (99,0-100,0)	100 (24,6)	75,7 (71,5-79,9)	110,0	216,7
Pentavalente (3ª dose)	98,2 (96,9-99,5)	141 (34,6)	65,0 (60,4-69,6)	108,8	170,1
Rotavírus (1ª dose)	95,3 (93,3-97,4)	22 (5,4)	89,8 (86,9-92,7)	75,8	128,2
Rotavírus (2ª dose)	92,0 (89,4-94,7)	54 (13,3)	79,2 (75,2-83,1)	37,0	70,0
Pneumocócica (1ª dose)	99,6 (99,0-100,0)	17 (4,2)	94,8 (92,7-97,0)	124,9	14,6
Pneumocócica (2ª dose)	98,3 (98,5-100,0)	47 (11,5)	87,6 (84,4-90,8)	171,9	276,9
Pneumocócica (dose de reforço)	96,6 (94,8-98,3)	154 (37,8)	57,1 (52,3-61,9)	89,5	144,5
Meningocócica (1ª dose)	99,6 (99,0-100,0)	66 (16,2)	83,7 (80,1-87,3)	108,1	248,3
Meningocócica (2ª dose)	98,5 (97,3-99,7)	118 (29,0)	70,0 (65,5-74,4)	94,3	167,5
Meningocócica (dose de reforço)	96,2 (94,4-98,1)	154 (37,8)	56,9 (52,0-61,7)	93,9	150,3
Febre amarela	95,5 (93,5-97,5)	148 (36,4)	58,8 (54,0-63,5)	177,7	244,4
SCR (1ª dose)	97,8 (96,4-99,2)	147 (36,1)	60,3 (55,5-65,0)	95,5	145,9
SCR (2ª dose)	96,4 (94,6-98,2)	173 (42,5)	52,7 (47,9-57,6)	125,1	163,9
Varicela (1ª dose)	95,5 (93,5-97,5)	169 (41,5)	53,9 (49,1-58,8)	132,9	177,1
Hepatite A	95,9 (94,0-97,8)	168 (41,3)	53,9 (49,1-58,8)	115,6	154,4
DTP (dose de reforço)	96,0 (94,1-97,9)	171 (42,0)	54,0 (49,2-58,9)	122,6	155,6
Poliomielite (dose de reforço)	96,3 (94,5-98,2)	179 (44,0)	54,2 (49,4-59,1)	126,3	160,5

DP: desvio apdrão; DTP: difteria, tétano e coqueluche; IC95%: intervalo de 95% de confiança; SCR: sarampo, caxumba e rubéola; VIP: vacina inativada da poliomielite.

* Atraso vacinal: doses recebidas após a idade limite para ser considerada correta, ou seja, 30 dias após o preconizado.

Em Cubatão, 86,9% das crianças nascidas nos anos de 2019 e 2020 tinham cobertura completa para o esquema vacinal básico (aos 12 meses). A proporção de crianças com cobertura completa variou entre os estratos socioeconômicos: o melhor desempenho no estrato 1, com 94,5% (IC95%: 87,5-97,7), e o pior no estrato 5, com 78,5% (IC95%: 73,1-83,0), e a diferença foi estatisticamente significativa (Tabela 4).

**Tabela 4:** Proporção de cobertura vacinal para o esquema básico completo aos 12 e 24 meses (doses aplicadas e doses sem atraso) e intervalo de 95% de confiança (IC95%) nas coortes de 2019 e 2020, por estrato socioeconômico. Inquérito de cobertura vacinal, Cubatão, São Paulo, Brasil, 2023.

Estrato socioeconômico (2019 e 2020)	Cobertura vacinal aos 12 meses		Cobertura vacinal aos 24 meses	
	Doses aplicadas	Doses sem atraso	Doses aplicadas	Doses sem atraso
	% (IC95%)	% (IC95%)	% (IC95%)	% (IC95%)
1	94,5 (89,3-99,7)	43,8 (32,5-55,2)	91,8 (85,5-98,1)	24,7 (14,8-34,5)
2	93,2 (87,4-98,9)	42,5 (31,1-53,8)	83,6 (75,1-92,1)	30,1 (19,6-40,7)
3	85,9 (78,5-93,3)	44,7 (34,1-55,3)	83,5 (75,6-91,4)	29,4 (19,7-39,1)
4	89,2 (82,5-95,8)	30,1 (20,3-40,0)	80,7 (72,2-89,2)	14,5 (6,9-22,0)
5	78,5 (70,1-86,8)	31,2 (21,8-40,6)	75,3 (66,5-84,0)	19,4 (11,3-27,4)
Total	86,9 (83,6-90,2)	39,9 (35,1-44,6)	82,6 (78,9-86,3)	25,4 (21,1-29,6)

Aos 24 meses, 82,6% das crianças tinham o esquema vacinal completo. A menor proporção foi observada no estrato 5 (84,5%; IC95%: 79,5-89,6) (Tabela 4).

Quando analisado esquema vacinal no momento oportuno, apenas 25,7% das crianças apresentaram esquema vacinal completo sem atraso. A maior proporção foi observada no estrato 2 (30,1%); e a menor proporção, no estrato 4 (14,5%) (Tabela 4).

Na análise univariada, atraso vacinal foi associado a residir em estratos de pior condição socioeconômica (estratos 4 e 5) (OR = 1,17; IC95%: 0,61-2,24), pertencer a um aglomerado intradomiciliar (OR = 1,29; IC95%: 0,44-3,80), criança do sexo masculino (OR = 1,20; IC95%: 0,67-2,12), criança de raça/cor da pele parda ou preta (OR = 1,42; IC95%: 0,73-2,78), mãe com trabalho remunerado (OR = 1,21; IC95%: 0,65-2,24), receber benefício do Bolsa Família (OR = 1,95; IC95%: 0,97-3,92) e mãe com 3 ou mais filhos vivos (OR = 1,54; IC95%: 0,82-2,88), porém nenhuma delas teve significância estatística. Por outro lado, as variáveis nível de consumo D (OR = 0,54; IC95%: 0,26-1,13), renda familiar acima de R$ 3.000,00 (OR = 0,60; IC95%: 0,30-1,22), frequentar creche (OR = 0,98; IC95%: 0,47-2,04), mãe com Ensino Médio completo ou Superior (OR = 0,32; IC95%: 0,14-0,75) e mãe de raça/cor da pele preta ou parda (OR = 0,85; IC95%: 0,43-1,70) estiveram associadas a esquema vacinal completo sem atraso aos 24 meses; e entre elas, apenas escolaridade da mãe teve significância estatística. Na análise multivariada, a escolaridade da mãe (OR = 0,32; IC95%: 0,15-0,68) e receber benefício do Bolsa Família (OR = 1,95; IC95%: 1,08-3,49) permaneceram independentemente associados ao atraso vacinal (Tabela 5).

**Tabela 5:** Razões de chances (OR) brutas e ajustadas e intervalos de 95% de confiança (IC95%) do atraso vacinal para esquema completo aos 24 meses pelas variáveis do estudo. Inquérito de cobertura vacinal, Cubatão, São Paulo, Brasil, 2023.

Variável	n (N = 407)	% com atraso vacinal	OR bruta (IC95%)	Valor de p	OR ajustada (IC95%)	Valor de p
Estrato						
1-3	231	69,3	1,00			
4-5	176	79,7	1,17 (0,61-2,24)	0,638		
Nível de consumo						
A-C	166	77,9	1,00			
D	194	77,3	0,54 (0,26-1,13)	0,103		
Renda familiar (R$)						
Até 3.000,00	261	85,3	1,00			
3.000,00 ou mais	113	65,9	0,60 (0,30-1,22)	0,161		
Escolaridade da mãe						
Até Ensino Médio incompleto	112	79,5	1,00		1,00	
Ensino Médio completo e Superior	258	134,1	0,32 (0,14-0,75)	0,008	0,32 (0,15-0,68)	0,003
Raça/Cor da pele da mãe						
Branco/Amarelo	139	69,8	1,00			
Preta/Parda	266	74,8	0,85 (0,43-1,70)	0,654		
Raça/Cor da pele da criança						
Branco/Amarelo	204	70,1	1,00			
Preta/Parda	202	78,2	1,42 (0,72-2,78)	0,300		
Filhos vivos						
1-2	266	70,9	1,00			
3 ou mais	140	70,6	1,54 (0,82-2,88)	0,177		
Aglomerado intradomiciliar						
Não	365	72,6	1,00			
Sim	42	83,3	1,30 (0,44-3,80)	0,638		
Recebe Bolsa Família						
Não	228	68,9	1,00		1,00	
Sim	177	79,7	1,95 (0,97-3,92)	0,059	1,95 (1,09-3,49)	0,025
Mãe com trabalho remunerado						
Não	190	75,8	1,00			
Sim	151	70,9	1,21 (0,65-2,24)	0,540		
Frequenta creche						
Não	91	76,9	1,00			
Sim	314	72,6	0,98 (0,47-2,03)	0,950		
Sexo da criança						
Feminino	184	72,8	1,00			
Masculino	223	74,4	1,20 (0,67-2,12)	0,538		

## Discussão

O inquérito domiciliar de cobertura vacinal para as coortes de crianças nascidas em 2019 e 2020 demonstrou o alcance das metas preconizadas pelo PNI para todas as vacinas do calendário de crianças menores de 2 anos de idade quando avaliadas individualmente (90% para BCG e rotavírus e 95% para as demais vacinas).

A cobertura do esquema vacinal completo aos 12 meses foi de 86,9%; e aos 24 meses foi de 82,6%. Todos os estratos socioeconômicos apresentaram cobertura para esquema completo abaixo do preconizado pelo PNI. Os estratos com pior condição socioeconômica tiveram as menores coberturas para o esquema completo aos 12 e aos 24 meses. Esses resultados sugerem menor acesso à vacinação das crianças residentes nas áreas mais empobrecidas da cidade.

Baixas coberturas vacinais são frequentemente relacionadas às condições geográficas, ao *status* socioeconômico da população ^21^, às características relacionadas às condições estruturais e de oferta e acesso aos serviços de saúde ^22^ ^,^ ^23^ e, mais recentemente, à pandemia da COVID-19 ^24^.

Cubatão tem 18 unidades básicas de saúde, no entanto quatro não dispõem de sala de vacinação. Entre as oito unidades localizadas nos estratos 4 e 5 (de menor condição socioeconômica), três não têm sala de vacina.

Embora estejam abaixo do preconizado, as coberturas para esquema vacinal completo encontradas neste estudo são maiores que as apresentadas em inquérito nacional realizado em 2020. Nas capitais dos estados brasileiros e no Distrito Federal a cobertura do esquema completo foi menor de 75%, variando de 74,4% em Curitiba a 35,8% em Macapá ^15^.

Desde 2019, uma das estratégias para elevar as coberturas vacinais adotadas em Cubatão é a obrigatoriedade de avaliação das carteiras de vacinação no momento da matrícula escolar. A apresentação de um atestado de calendário vacinal completo para a idade emitido por uma unidade de saúde é exigida para ingressar nas escolas municipais. Isso pode explicar em parte a maior cobertura vacinal na cidade, visto que 77,1% das crianças do estudo frequentam creche ou escola, o que pode ter contribuído para a regularização das doses. Entre as crianças que frequentam a creche, a cobertura vacinal completa aos 24 meses foi de 87% (273/314); e para as que não frequentam foi de 68,1% (62/91).

Embora a maior parte das crianças tenha recebido as doses previstas até 24 meses de vida (82,6%), apenas 23,2% receberam essas vacinas no momento oportuno. Resultado semelhante ao observado no Maranhão onde 23,6% das crianças não estavam adequadamente vacinadas aos 12 meses de idade ^18^.

A avaliação de doses aplicadas de maneira oportuna sem atraso tem sido relatada como um indicador crucial para compreender a suscetibilidade da doença em cada nível populacional ^25^ ^,^ ^26^. Estudos em países de alta renda têm revelado que apesar das elevadas coberturas vacinais, grande proporção das crianças recebe as vacinas depois da idade recomendada ^27^. Inquérito nacional conduzido nos Estados Unidos ^28^ encontrou cobertura vacinal de 73% para crianças entre 19 e 35 meses de idade, mas somente 30% dessas crianças haviam recebido todas as vacinas na idade recomendada durante os primeiros 24 meses de vida.

Neste estudo observou-se que quanto maior a idade recomendada para aplicação do imunobiológico, maior a proporção de crianças com atraso vacinal. Esse dado é similar ao encontrado no Canadá, onde a proporção de atraso aumentou de 5,4% aos 2 meses de idade para 23,6% aos 12 meses ^29^, e no Brasil onde há queda progressiva da cobertura para todas as vacinas que exigem mais de uma dose ^15^.

O momento da vacinação (dose sem atraso) permite avaliar o processo de trabalho das salas de vacinação, uma vez que os programas de imunização só podem ser bem-sucedidos se as crianças estiverem protegidas antes da exposição ^30^. Uma das ações prioritárias dos serviços com salas de vacina deve ser elaborar estratégias para atualização da vacinação, realizando, por exemplo, avaliação das carteiras de vacinação nas visitas domiciliares, nos comparecimentos à unidade de saúde para outras atividades e na busca de faltosos. Outro aspecto observado nos dados deste inquérito é que algumas vacinas que devem ser aplicadas na mesma data (por exemplo, 2ª dose de pneumocócica e 2ª dose de pentavalente) apresentam cobertura vacinal diferente sugerindo que, embora a criança tenha comparecido à sala de vacinação para atualização, algumas doses não foram aplicadas.

É importante conhecer as características das crianças que têm maior probabilidade de sofrer atrasos vacinais para formular estratégias para redução de vacinação inoportuna ^31^. Em Rondonópolis, Mato Grosso, o atraso para o esquema completo aos 12 meses foi associado a ter um irmão ou mais no domicílio e não receber visita de agente comunitário de saúde nos últimos 30 dias ^4^. No Maranhão, o atraso foi associado a chefes de família das classes econômicas menos favorecidas, do sexo feminino e de raça/cor da pele preta ^18^.

Nossos resultados mostraram associação de atraso vacinal com menor escolaridade da mãe e receber benefício do Bolsa Família. A baixa escolaridade materna como fator de risco para atraso vacinal tem sido controversa na literatura ^32^: alguns estudos encontram essa associação ^29^ ^,^ ^33^, enquanto em outros o atraso esteve associado ao elevado nível educacional da mãe ^34^. Em uma revisão da literatura, o nível educacional elevado foi associado à hesitação em vacinar, o que pode explicar o atraso vacinal nessa população ^35^.

Já a associação entre baixo nível educacional e atraso vacinal tem sido explicada na literatura pela menor compreensão dos benefícios da vacinação e da dificuldade de comunicação entre o profissional de saúde e os cuidadores ^36^.

O Bolsa Família é um programa federal de transferência de renda destinado a famílias em situação de pobreza. Uma das condicionalidades para manter o benefício é o cumprimento do Calendário Nacional de Vacinação da criança menor de 7 anos de idade. Inquérito domiciliar realizado em Salvador (Bahia) mostra que crianças que pertencem a famílias que recebem o benefício têm maior chance de apresentar a carteira atualizada ^37^. No entanto, estudos realizados em outras regiões do país não encontraram os mesmos resultados ^38^ ^,^ ^39^. Em São Paulo e Maranhão, a incompletude vacinal é maior nas crianças de baixa renda, e receber o benefício do programa não teve influência sobre a vacinação infantil. Os autores sugerem que a condicionalidade não está sendo observada adequadamente, ou talvez somente ela, isoladamente, não seja suficiente para garantir a cobertura vacinal se outras ações não forem implementadas, tais como a ampliação do acesso à atenção primária e a garantia de disponibilidade contínua de vacinas nas unidades básicas, sem interrupções ^39^.

No nosso estudo, entre as crianças com pior condição socioeconômica (estrato 4 e 5), a cobertura vacinal de doses sem atraso para as beneficiárias do Bolsa Família foi de 17,5%, enquanto para as não beneficiárias foi de 18,8%, indicando que o programa pode não ter impacto no atraso vacinal na população mais vulnerável.

Diante da pandemia de COVID-19, o governo brasileiro criou, em 2020, um benefício (Auxílio Emergencial) com o objetivo de garantir renda mínima aos brasileiros em situação de vulnerabilidade. O valor mensal desse benefício era superior ao do Bolsa Família e não foi permitido acumular os dois benefícios. Além disso, o Auxílio Emergencial não incluía condicionalidades de saúde para manutenção dos repasses ^40^. Isso pode ter contribuído para que o acompanhamento de vacinação tenha sido menos exigido nos anos de pandemia quando as crianças que fazem parte do estudo estavam em idade de completar o calendário vacinal de menores de 2 anos.

Este estudo apresenta algumas limitações. Apenas crianças com carteira de vacinação no momento da entrevista participaram do estudo, o que pode ter contribuído para superestimar os dados de cobertura, visto que ter carteira de vacinação pode estar associado a maior probabilidade de estar imunizado. Outra limitação é que as informações de imunização e sociodemográficas como Bolsa Família, aglomeração domiciliar e renda foram coletadas no mesmo momento, o que pode dificultar o estabelecimento de associação entre essas características e o status vacinal da criança.

Este inquérito avaliou o estado de vacinação de crianças aos 12 e aos 24 meses de idade em um município de aproximadamente 112 mil habitantes no litoral de São Paulo e revelou que os atrasos antes de completar o esquema de vacinação infantil são muito frequentes. As informações apresentadas revelam que, embora a cobertura vacinal seja maior do que a observada no inquérito nacional, o atraso para aplicação das doses é um problema de saúde pública em Cubatão, o que exige novas intervenções, além de educação dos profissionais de saúde da ponta e da população sobre a importância da vacinação em tempo oportuno.

## References

[1] Andrade MV, Noronha K, Cardoso CS, Oliveira CL, Calazans JA, Souza MN (2021). Análise da concordância entre as informações reportadas pelas mães e dos cartões de vacina das crianças no Brasil (2013 e 2015). Cad Saúde Colet (Rio J.).

[2] Ministério da Saúde (2003). Programa Nacional de Imunizações: 30 anos.

[3] Sato APS (2018). What is the importance of the vaccine hesitancy in the drop of vaccination coverage in Brazil. Rev Saúde Pública.

[4] Lemos PL, Takano OA (2022). Factors associated with the incomplete opportune vaccination schedule up to 12 months of age, Rondonópolis, Mato Grosso. Rev Paul Pediatr.

[5] Arroyo LH, Ramos ACV, Yamamura M, Weiller TH, Crispim JA, Cartagena-Ramos D (2020). Áreas com queda da cobertura vacinal para BCG, poliomielite e tríplice viral no Brasil (2006-2016) mapas da heterogeneidade regional. Cad Saúde Pública.

[6] Buffarini R, Barros FC, Silveira MF (2020). Vaccine coverage within the first year of life and associated factors with incomplete immunization in a Brazilian birth cohort. Arch Public Health.

[7] Pacheco FC, França GVA, Elidio GA, Domingues CMAS, de Oliveira C, Guilhem DB (2019). Trends and spatial distribution of MMR vaccine coverage in Brazil during 2007-2017. Vaccine.

[8] Césare N, Mota TF, Lopes FFL, Lima ACM, Luzardo R, Quintanilha LF (2020). Longitudinal profiling of the vaccination coverage in Brazil reveals a recent change in the patterns hallmarked by differential reduction across regions.. Int J Infect Dis.

[9] Silveira MF, Tonial CT, Goretti K, Maranhão A, Teixeira AMS, Hallal PC (2021). Missed childhood immunizations during the COVID-19 pandemic in Brazil analyses of routine statistics and of a national household survey. Vaccine.

[10] Souza JFA, Silva TPR, Silva TMR, Amaral CD, Ribeiro EEN, Vimieiro AM (2022). Cobertura vacinal em crianças menores de um ano no estado de Minas Gerais, Brasil. Ciênc Saúde Colet.

[11] Moraes JC, Ribeiro MC (2008). Desigualdades sociais e cobertura vacinal uso de inquéritos domiciliares. Rev Bras Epidemiol.

[12] Mota E (2008). Inquérito domiciliar de cobertura vacinal a perspectiva do estudo das desigualdades sociais no acesso à imunização básica infantil. Rev Bras Epidemiol.

[13] Dombkowski KJ, Lantz PM, Free GL (2002). The need for surveillance of delay in age-appropriate immunization. Am J Prev Med.

[14] Dayan GH, Shaw KM, Baughman AL, Orellana LC, Forlenza R, Ellis A (2006). Assessment of delay in age-appropriate vaccination using survival analysis. Am J Epidemiol.

[15] Barata RB, França AP, Guibu IA, Vasconcellos MTL, Moraes JC, Grupo ICV 2020 (2023). National Vaccine Coverage Survey 2020: methods and operational aspects.. Rev Bras Epidemiol.

[16] Barata RB, Pereira SM (2013). Desigualdades sociais e cobertura vacinal na cidade de Salvador, Bahia. Rev Bras Epidemiol.

[17] Queiroz LLC, Monteiro SG, Mochel EG, Veras MASM, Sousa FGM, Bezerra MLM (2013). Cobertura vacinal do esquema básico para o primeiro ano de vida nas capitais do Nordeste brasileiro. Cad Saúde Pública.

[18] Yokokura AVCP, Silva AAM, Bernardes ACF, Lamy F, Alves MTSSB, Cabra NAL (2013). Cobertura vacinal e fatores associados ao esquema vacinal básico incompleto aos 12 meses de idade, São Luís, Maranhão, Brasil, 2006. Cad Saúde Pública.

[19] Dean AG, Sullivan KM, Soe MM OpenEpi: open source epidemiologic statistics for public health..

[20] Tauil MC, Sato APS, Costa ÂA, Inenami M, Ferreira VLR, Waldman EA (2017). Coberturas vacinais por doses recebidas e oportunas com base em um registro informatizado de imunização, Araraquara-SP, Brasil, 2012-2014. Epidemiol Serv Saúde.

[21] Bates AS, Wolinsky FD (1998). Personal, financial, and structural barriers to immunization in socioeconomically disadvantaged urban children. Pediatrics.

[22] Guzman-Holst A, DeAntonio R, Prado-Cohrs D, Juliao P (2020). Barriers to vaccination in Latin America a systematic literature review. Vaccine.

[23] Figueiredo A, Johnston IG, Smith DMD, Agarwal S, Larson HJ, Jones NS (2016). Forecasted trends in vaccination coverage and correlations with socioeconomic factors a global time-series analysis over 30 years. Lancet Glob Health.

[24] Bramer CA, Kimmins LM, Swanson R, Kuo J, Vranesich P, Jacques-Carroll LA (2022). Decline in child vaccination coverage during the COVID-19 pandemic - Michigan Care Improvement Registry, May 2016-May 2020. MMWR Morb Mortal Wkly Rep.

[25] Mutua MK, Kimani-Murage E, Ngomi N, Ravn H, Mwaniki P, Echoka E (2016). Fully immunized child coverage, timing and sequencing of routine immunization in an urban poor settlement in Nairobi Kenya. Trop Med Health.

[26] Fadnes LT, Nankabirwa V, Sommerfelt H, Tylleskär T, Tumwine JK, Engebretsen IM (2011). Is vaccination coverage a good indicator of ageappropriate vaccination A prospective study from Uganda. Vaccine.

[27] Hull BP, McIntyre PB (2006). Timeliness of childhood immunisation in Australia. Vaccine.

[28] Luman ET, Barker LE, Shaw KM, McCauley MM, Buehler JW, Pickering LK (2005). Timeliness of childhood vaccinations in the United States days undervaccinated and number of vaccines delayed. JAMA.

[29] Kiely M, Boulianne N, Talbot D, Ouakki M, Guay M, Landry M (2018). Impact of vaccine delays at the 2, 4, 6 and 12 month visits on incomplete vaccination status by 24 months of age in Quebec, Canada. BMC Public Health.

[30] Scott S, Odutola A, Mackenzie G, Fulford T, Afolabi MO, Lowe Jallow Y (2014). Coverage and timing of children&apos;s vaccination an evaluation of the expanded programme on immunisation in The Gambia. PLoS One.

[31] Dombkowski KJ, Lantz PM, Freed GL (2004). Risk factors for delay in age-appropriate vaccination. Public Health Rep.

[32] Sisson H, Gardiner E, Watson R (2017). Vaccination timeliness in preterm infants an integrative review of the literature. J Clin Nurs.

[33] Gebremeskel TG, Hagos MG, Kassahun SS, Gebrezgiher BH (2021). Magnitude and associated factors of delayed vaccination among children aged 11-23 months in Tigray, Ethiopia, 2018. Hum Vaccin Immunother.

[34] Alam MJ, Afsar MNA, Khanam A, Ahmad SM (2021). Risk factors for delay in starting age-appropriate vaccinations among infants in urban slums of Bangladesh. Hum Vaccin Immunother.

[35] Larson HJ, Jarrett C, Eckersberger E, Smith DM, Paterson P (2014). Understanding vaccine hesitancy around vaccines and vaccination from a global perspective a systematic review of published literature, 2007-2012. Vaccine.

[36] Negash BT, Tediso Y, Yoseph A (2023). Predictors of timeliness of vaccination among children of age 12-23 months in Boricha district, Sidama region Ethiopia, in 2019. BMC Pediatr.

[37] Shei A, Costa F, Reis MG, Ko AI (2014). The impact of Brazil´s Bolsa Família conditional cash transfer program on children´s health care utilization and health outcomes.. BMC Int Health Hum Rights.

[38] Andrade MV, Chein F, Souza LR, Puig-Junoy J (2012). Income transfer policies and the impacts on the immunization of children the Bolsa Família Program. Cad Saúde Pública.

[39] Silva FS, Queiroz RCS, Branco MRFC, Simões VMF, Barbosa YC, Rodrigues MAFRA (2020). Programa Bolsa Família e vacinação infantil incompleta em duas coortes brasileiras. Rev Saúde Pública.

[40] Brasil (2020). Decreto nº 10.316, de 7 de abril de 2020. Regulamenta a Lei nº 13.982, de 2 de abril de 2020, que estabelece medidas excepcionais de proteção social a serem adotadas durante o período de enfrentamento da emergência de saúde pública de importância internacional decorrente do coronavírus (COVID-19).. Diário Oficial da União.

[41] Associação Brasileira de Empresas de Pesquisa Critério de classificação econômica Brasil. Versão 2021..

